# P-623. Multicentric Prospective Study on Invasive Pneumococcal Disease (IPD) and Community Acquired Pneumonia (CAP) in hospitalized children, Serotype distribution and Antimicrobial Susceptibility Test (ATS) in Argentina after 11 years of Pneumococcal Conjugated Vaccine (PCV13) in National Immunization Program (NIP)

**DOI:** 10.1093/ofid/ofae631.821

**Published:** 2025-01-29

**Authors:** Eduardo L López, Valeria Uriarte, Mariel García, Andres Gomila, Gustavo Cesar Ezcurra, Lola Vozza, Liliana Bertoni, Adrián Morales, Eduardo Teplitz, Claudia Diaz Argüello, Clarisa Aguirre, Claudia Lara, Alejandra Corso, María M Contrini

**Affiliations:** Pediatric Infectious Disease Program, Hospital de Niños Ricardo Gutiérrez, Universidad de Buenos Aires, Ciudad Autónoma de Buenos Aires, Buenos Aires, Argentina; Hospital de Niños "Sor María Ludovica ", La Plata, Buenos Aires, Argentina; Hospital de Niños "Sor María Ludovica", La Plata, Buenos Aires, Argentina; Hospital de Niños "Santísima Trinidad", Córdoba, Cordoba, Argentina; Hospital Orlando Alassia, Santa Fe, Santa Fe de la Vera Cruz, Santa Fe, Argentina; Hospital "Nuevo Siglo", Cordoba, Cordoba, Argentina; Hospital "Guillermo Rawson", San Juan, San Juan, Argentina; Hospital Provincial "Castro Rendon", Neuquen, Neuquen, Argentina; Hospital Italiano de Bahía Blanca, Bahía Blanca, Buenos Aires, Argentina; Hospital Italiano de Bahía Blanca, Bahía Blanca, Buenos Aires, Argentina; Hospital de Niños "San Juan Pablo II", Corrientes, Corrientes, Argentina; Instituto Nacional de Enfermedades Infecciosas ANLIS "Dr Carlos G Malbran", Ciudad Autónoma de Buenos Aires, Ciudad Autonoma de Buenos Aires, Argentina; Instituto de Enfermedades Infecciosas ANLIS "Dr Carlos G Mabrán", Buenos Aires, Buenos Aires, Argentina; Pediatric Infectious Disease Program, Hospital de Niños Ricardo Gutiérrez, Universidad de Buenos Aires, Ciudad Autónoma de Buenos Aires, Buenos Aires, Argentina

## Abstract

**Background:**

PCV13 was included in Argentina NIP in 2012 with 2 +1 schedule at 2, 4 and 12-15 mo of age. After 11 yrs program, from May´23-Mar´24 we carried out a multicentric prospective study to evaluate impact of pneumococcal disease, *S.pneumoniae* (*Spn*) serotype(S) distribution and AST.

**Methods:**

Study Population: 1 mo to 10 yr old hospitalized children by IPD and/or CAP at 8 pediatric centers; f-up 6 mo. Clinical record was completed for subjects who met inclusion criteria after written Inform Consent from parents/legal guardian was obtained. *Spn* isolates from normally sterile fluids were sent to National Reference Laboratories for serotyping by Quellung reaction and AST by agar dilution (CSLI 2024).

**Results:**

Subjects: 149; mean age(±SD): 49.2(±34) mo; male 83(55.7%). Age distribution: <1yr: 23(15.4%); 1-2 yr: 36(24.1%); 3-5 yr: 49(32.9%); >5 yr: 41(27.5%). Two main risk factors for IPD/CAP were: smoker at home 51(34.2%) and daycare attendance 41(30.2%). Diagnosis on admission: CAP 97(65.1%); pneumonia with empyema 54(36.2%); bacteremia 22(14.8%); meningitis 7(4.7%); peritonitis 5(3.4%); other 13(8.7%). *Spn* vaccination status was verified in 137/149(90.5%) subjects; being complete by age in: <1yr: 65.2%; 1-2yr: 80.6%; 3-5yr: 89.8%; >5yr: 87.8% subjects. Among 40 *Spn* the most prevalent S (72.5%) were (n): 12F(6), 3(4), 19A(3), 24B(3), 38(3), 24F(2), 23B(2), 1(2), 10A(2) and 11A(2). Other S were: 13, 14, 34, 15A, 15B, 16F, 17F, 22F, 23A, 7C and 9N. % No-susceptibility (NS) was: penicillin (PEN) meningitis/no meningitis 35/5, cefotaxime meningitis/no meningitis 7.5/0. All were 100% susceptible to rifampicin, vancomycin, ceftaroline and ceftobiprole. NS-PEN (CIM ≥0.12ug/ml) was associated with S 24B, 24F, 19A, 23B (71.4%). S coverage was 25%(10/40) for PCV13 (S 3, 19A and 14); and 55%(22/40) for PCV20 (S 12F, 3, 19A, 10A, 11A, 14, 15B and 22F).

**Conclusion:**

1. Children < 1yr old have the highest rate of incomplete *Spn* vaccination rate,then it is needed to improve the vaccine coverage in this age group in Argentina

2. Our preliminary data show an estimated coverage of 25% with PCV13 and 55% with PCV20

3. According to *Spn* S detected in IPD and CAP, PCV20 could increase the coverage by 30% in children in Argentina

**Disclosures:**

**All Authors**: No reported disclosures

